# Reply to: “Vitamin D Insufficiency May Account for Almost Nine of Ten COVID-19 Deaths: Time to Act. Comment on: Vitamin D Deficiency and Outcome of COVID-19 Patients. *Nutrients* 2020, *12*, 2757”

**DOI:** 10.3390/nu12123643

**Published:** 2020-11-27

**Authors:** Aleksandar Radujkovic, Uta Merle

**Affiliations:** 1 Department of Internal Medicine V, University of Heidelberg, 69120 Heidelberg, Germany; aleksandar.radujkovic@med.uni-heidelberg.de; 2 Department of Internal Medicine IV, University of Heidelberg, 69120 Heidelberg, Germany

We thank Brenner and Schöttker for their comment [1] and interest in our recent publication [2] and for providing us with the opportunity to extend the discussion regarding our observations and the potential role of vitamin D (VitD) supplementation in reducing disease severity and the risk of mortality in individuals infected with SARS-CoV-2.

As already pointed out and discussed [2], the results of observational studies always need to be interpreted with caution, since they are vulnerable to bias and unknown confounders. Although a covariate-adjusted hazard of death of ~11 for VitD insufficient patients (25(OH)D <20 ng/mL at baseline) was observed, it should be noted that due to the small sample-size and particularly the low number of death events, we were only able to include a limited number of confounding variables (age, gender, and presence of any comorbidity) in our models. However, as already pointed out [2], the presence of additional confounding risk factors for COVID-19 severity, such as obesity or specific comorbidities, as well as other unrecognized factors, should also be borne in mind, in particular since many of these at-risk patients are also disproportionally affected by poor VitD status. In other words, in an observational study design, it cannot be excluded that VitD deficiency represents a surrogate marker for a general micronutrient deficiency, which in turn reflects only the patient’s overall health status. For instance, obesity, which is associated with chronic low-grade inflammation and higher IL-6 levels and risk of hospitalization from respiratory tract infections, was recently also shown to be a determinant of COVID-19 severity and mortality [3,4,5]. In addition, although evidence is accumulating that suggests COVID-19 mortality to be associated with poor VitD status [6,7,8], studies which found no association with disease outcomes [9] or mortality [10] also need to be acknowledged. Therefore, in the absence of a randomized controlled trial on VitD treatment, no causal association between VitD status and severity/outcome of COVID-19 can be inferred. On the other hand, as rightly pointed out by Brenner and Schöttker [1], results of such trials, particularly the large VIVID trial [11], will not be available in the near future.

In general, as in all treatment decisions, the potential benefits of therapy must outweigh the risks. We agree with Brenner and Schöttker that in the case of VitD, the risk–benefit ratio is probably skewed far in favor of benefits. Therefore, pending randomized controlled trial evidence, and facing an emerging second wave of SARS-CoV-2 infections, it would seem uncontroversial to promote efforts to achieve sufficient 25(OH)D levels, particularly for high-risk groups where VitD deficiency is highly prevalent, and advocate for targeted VitD supplementation for all SARS-CoV-2 infected individuals.
